# Sugarloaf Land in south-eastern Brazil: a tropical hotspot of lowland inselberg plant diversity

**DOI:** 10.3897/BDJ.8.e53135

**Published:** 2020-06-19

**Authors:** Luiza F. A. de Paula, Luísa O. Azevedo, Luana Paula Mauad, Leandro Jorge Telles Cardoso, João Marcelo Alvarenga Braga, Ludovic J.C. Kollmann, Claudio N. Fraga, Luiz Menini Neto, Paulo H. Labiak, Renato Mello-Silva, Stefan Porembski, Rafaela Campostrini Forzza

**Affiliations:** 1 Universidade Federal de Minas Gerais, Belo Horizonte, Brazil Universidade Federal de Minas Gerais Belo Horizonte Brazil; 2 Jardim Botânico do Rio de Janeiro, Rio de Janeiro, Brazil Jardim Botânico do Rio de Janeiro Rio de Janeiro Brazil; 3 Instituto Nacional da Mata Atlântica, Museu de Biologia Prof. Mello Leitão, Santa Teresa, Brazil Instituto Nacional da Mata Atlântica, Museu de Biologia Prof. Mello Leitão Santa Teresa Brazil; 4 Universidade Federal de Juiz de Fora, Juiz de Fora, Brazil Universidade Federal de Juiz de Fora Juiz de Fora Brazil; 5 Universidade Federal do Paraná, Curitiba, Brazil Universidade Federal do Paraná Curitiba Brazil; 6 Universidade de São Paulo, São Paulo, Brazil Universidade de São Paulo São Paulo Brazil; 7 Universität Rostock, Rostock, Germany Universität Rostock Rostock Germany

**Keywords:** Atlantic Forest, granite outcrops, rock outcrops, rupicolous plants

## Abstract

**Background:**

Isolated monoliths of granitic and/or gneissic rock rising abruptly from the surrounding landscape are known as inselbergs. Dome-shaped inselbergs are common throughout the Atlantic Forest in south-eastern Brazil, a region known as Sugarloaf Land (SLL). This study aimed to create the first checklist of vascular plant species occurring on lowland inselbergs in SLL, with a focus on vegetation islands. We used information from online databases, our own field sampling and data from previously-published studies. We found 548 vascular plant species (505 angiosperms; 43 ferns and lycophytes) belonging to 69 families and 212 genera. Of all identified species, 536 are native and 12 are naturalised.

**New information:**

We updated the information currently available in Flora do Brasil 2020, as 59% of the angiosperms and 63% of the ferns and lycophytes on our checklist were not previously characterised as occurring on rock outcrops. As a first step towards generating a Virtual Herbarium of lowland inselberg vascular plants, we added barcode vouchers with images available online for 75% of the total number of vascular species. In the official lists of endangered species, 115 angiosperms and five ferns and lycophytes are mentioned. However, the conservation status of many species have not yet been evaluated (77% angiosperms; 88% ferns and lycophytes), thus this list is an important step towards their conservation. The information provided herein is essential for management programmes related to rock outcrops in Brazil as they are facing serious threats to conservation.

## Introduction

Brazil contains the richest seed plant diversity in the world (BFG 2018). Surprisingly, inventories in well-studied areas, such as the Atlantic Forest in south-eastern (SE) Brazil, are still documenting new records and identifying species new to science (Sobral and Stehmann 2009). This is especially true for overlooked habitats within this domain, such as inselbergs (Barthlott and Porembski 2000), isolated monoliths of granitic and/or gneissic rock, where dozens of new species belonging to different families have been identified over the last decade (e.g. Kollmann and Fontana 2010, Leme et al. 2010a, Leme et al. 2010b, Viana and de Paula 2013, Fraga and Guimarães 2014, de Oliveira and Sobrado 2016, Gonçalves and de Paula 2016, Gouvêa et al. 2018, Meyer et al. 2018, Fraga et al. 2019, Morales and Kollmann 2019, Valadares et al. 2019). These ancient rock outcrops are common elements in various landscapes around the world (Hopper et al. 2016) and are especially biodiverse in SE Brazil, the central highlands of Madagascar and in south-western Australia (Porembski 2007). They are characterised by extreme edaphic and microclimatic conditions, leading to their ecological isolation from the surrounding matrix and their island-like characteristics (Porembski et al. 2000). Consequently, their vegetation is distinct from the surrounding area (Parmentier et al. 2005, Porembski 2007).

In many parts of Brazil, inselbergs are characteristic elements of the landscape, particularly in the Atlantic Forest domain where they occur in large numbers and at varying sizes, altitudes and degrees of isolation (Safford and Martinelli 2000). The term “Sugarloaf” (*pão de açúcar*) is associated with lowland, isolated and dome-shaped inselbergs in the rainforest in SE Brazil (Ab’Sáber 1967). As the core area of lowland inselbergs seems to form a particular phytogeographical region, it has been named Sugarloaf Land (de Paula et al. 2016). Literature on the flora and vegetation of inselbergs is sparse and data on their ecological characteristics are mostly descriptive. Despite the lack of detailed knowledge about Brazilian inselbergs, previous regional studies have suggested extraordinarily high floristic richness (Meirelles et al. 1999, Safford 1999, Caiafa and Silva 2005, Ribeiro et al. 2007, Couto et al. 2017) which is further corroborated by the occurrence of high levels of beta diversity amongst inselbergs from SE Brazil (Meirelles et al. 1999, Safford and Martinelli 2000, de Paula et al. 2019b). It is also common for inselberg plant communities to be endemic to a specific region, often with species isolated to a single outcrop (de Paula et al. 2016, de Paula et al. 2017a, Couto et al. 2017). Additionally, recent phylogeographic studies show that inselberg endemics display strong genetic differentiation amongst individual rock outcrops, indicating low degrees of gene flow (e.g. *Palma-Silva et al. 2011, Hmeljevski et al. 2015, Hmeljevski et al. 2017*).

Although no specific reference to mountains, including rock outcrops, exists in Brazilian legislation on biodiversity (Martinelli 2007), these environments have been highlighted as an important aspect of the Convention on Biological Diversity (2002), in which the Mountain Work Programme (MWP) was proposed to reduce global, regional and local loss of mountain biodiversity (Martinelli 2007). The lack of specific legislation has resulted in significant gaps in knowledge related to the flora of several mountain regions throughout Brazil (Safford and Martinelli 2000, Carmo et al. 2018). This scenario is worrying since inselbergs and rock outcrops, in general, are amongst the most threatened and neglected environments in the world (Porembski et al. 2016).

Considering the lack of research on, and rapid destruction of, these unique landscape features and, in order to pay particular attention to the high levels of biodiversity in these ecotonal habitats, this study presents a list of vascular plants occurring on lowland inselbergs in the Atlantic Forest in SE Brazil. We aim to provide comprehensive and updated information regarding taxonomic nomenclature, life forms and conservation status, while also illustrating the unique diversity of Sugarloaf Land.

## Project description

### Study area description

**Figure 1.**
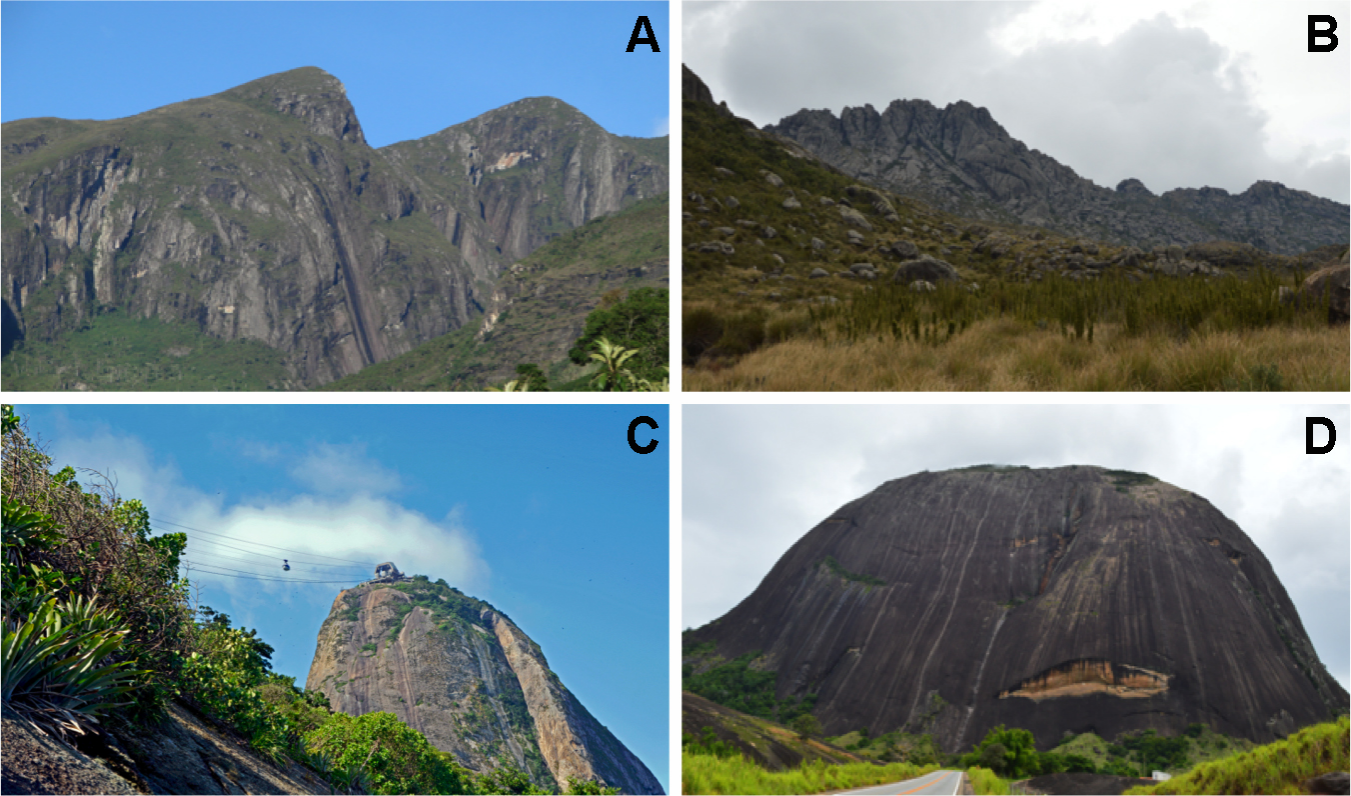
Examples of highland and lowland inselbergs occurring in SE Brazil. The first row represents highland inselbergs; A. Serra do Caparaó, Minas Gerais State; B. Serra do Itatiaia, Rio de Janeiro State. The second row represents lowland inselbergs, also known as sugarloaves; C. Sugarloaf Mountain, Rio de Janeiro; D. Pedra da Boca, Minas Gerais. Photos by L.F.A. de Paula, except for A. by N.F.O. Mota.

Inselbergs are found in large concentrations in eastern Brazil (Vieira et al. 2015). In SE Brazil in particular, two main types of granite outcrops occur: highland and lowland inselbergs (Safford and Martinelli 2000). Highland inselbergs are known as *campos de altitude* (Brazilian Highlands, *sensu* Safford 1999) and can be found in the mountain ranges of Serra da Mantiqueira and Serra do Mar, as well as in their subranges and disjunctions, such as Serra do Itatiaia, Serra dos Órgãos, Serra do Brigadeiro and Serra do Caparaó (Fig. 1, A-B). On the other hand, lowland inselbergs are a group of dome-shaped monolithic outcrops with a sugarloaf morphology (Ab’Sáber 1967, Fig. 1, C-D), the most iconic example of which is Sugarloaf Mountain (*Pão de Açúcar*) in Rio de Janeiro. The core area encompassing the states of Rio de Janeiro, Espírito Santo, southern Bahia and the adjacent region in Minas Gerais, were recently denominated Sugarloaf Land due to the concentration of lowland inselbergs that harbour high levels of plant species richness and elevated rates of endemism (de Paula et al. 2016). However, estimates of diversity in this area have been based solely on local inventories or on surveys of specific taxonomic plant groups (e.g. *Porembski et al. 1998, Meirelles et al. 1999, de Paula et al. 2016, de Paula et al. 2017a*).

Highland and lowland inselbergs have distinct floristic composition (Safford 1999, Safford and Martinelli 2000). The present checklist focuses on the lowland inselbergs from Sugarloaf Land (de Paula et al. 2016), which are defined as dome-shaped with isolated peaks at an elevation of less than 1000 m above sea level (a.s.l.). Highland inselbergs were not considered in the present study, nor were transitional areas where most inselbergs are located above 1000 m a.s.l., but not considered *campos de altitude*, such as Pedra Azul, Forno Grande, Alto Misterioso (Espírito Santo State) and Pico da Caledônia (Rio de Janeiro State). We also excluded rocky shores (*costões rochosos*), inselbergs located in domains other than the Atlantic Forest biome and other types of rock outcrops, such as *campos rupestres* (*sensu Silveira et al. 2016*), karstic outcrops (*sensu Bystriakova et al. 2019*) and *cangas* (*sensu* Carmo et al. 2018).

## Sampling methods

### Study extent

***Vascular plant dataset***

**Species list compilation**: We obtained a list of Brazilian angiosperms from Brazilian Flora Group (BFG 2015) and a list of Brazilian ferns and lycophytes from Prado et al. (2015). To filter the species unique to Sugarloaf Land, we created a protocol (procedures 1 and 2) to clean the datasets.

**Figure 2.**
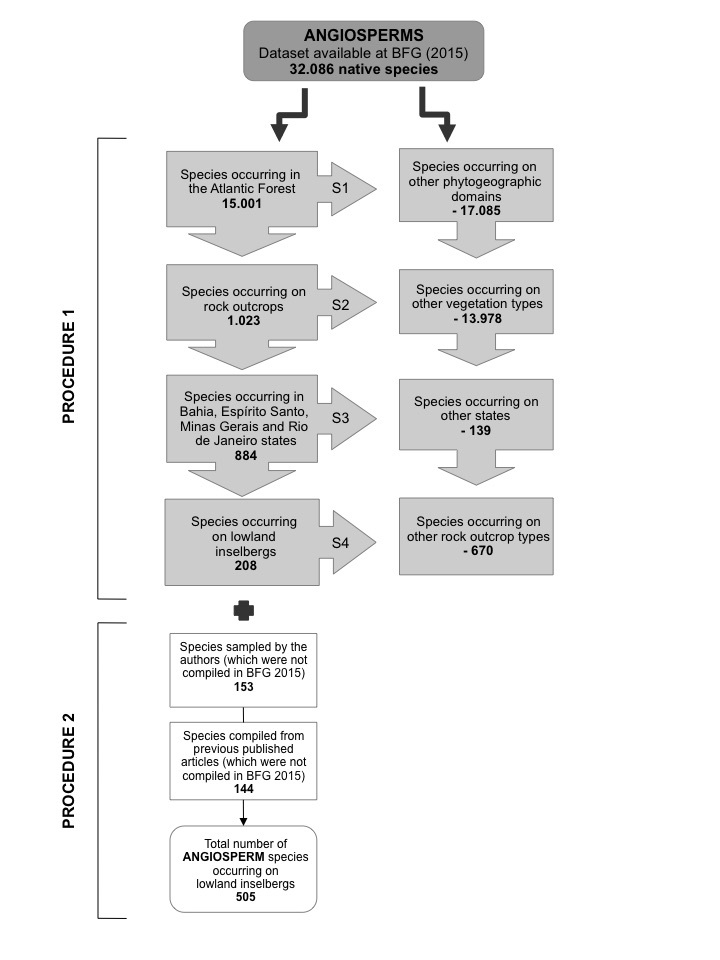
Procedure 1: stages of data filtering to obtain the angiosperm species list for lowland inselbergs, SE Brazil, based on the list available from BFG (2015). The left column shows species that remained during the construction and validation of the list and the right column represents species removed from the list; S1-S4: indicates steps 1- 4 (see text for more details). Procedure 2: stages of data addition to obtain the final angiosperm species list for lowland inselbergs, SE Brazil.

**Figure 3.**
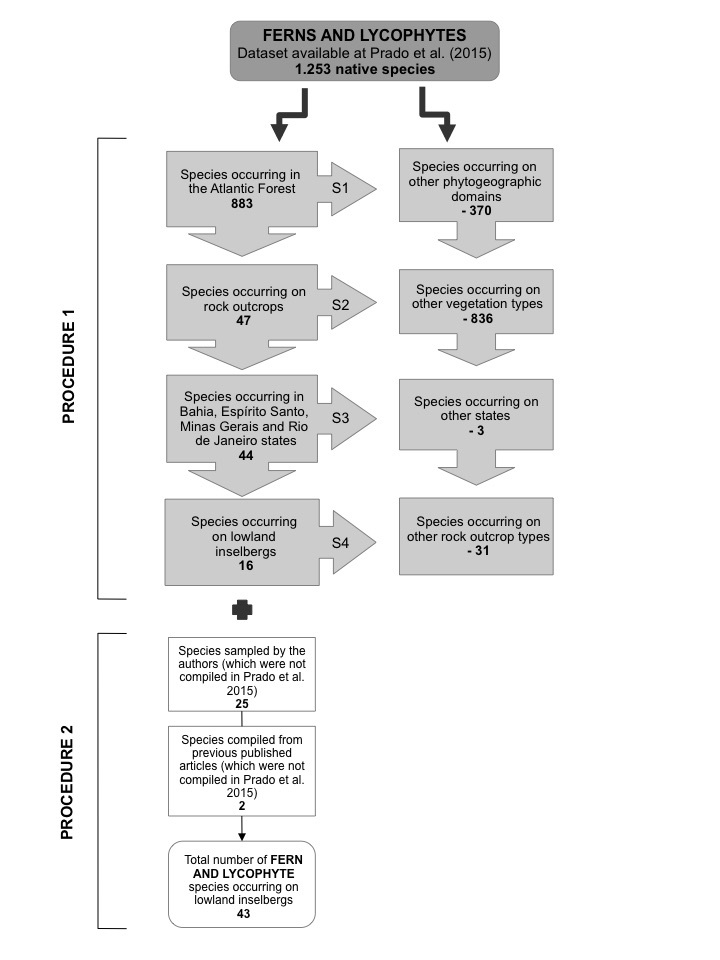
Procedure 1: stages of data filtering to obtain the fern and lycophyte species list from lowland inselbergs, SE Brazil, based on the list available from Prado et al. (2015). The left column represents species that remained during the construction and validation of the list and the right column represents species removed from the list; S1-S4: indicates steps 1- 4 (see text for more details). Procedure 2: stages of data addition to obtain the final fern and lycophyte species list for lowland inselbergs, SE Brazil.

In **procedure 1**, four steps were conducted separately for the angiosperm (Fig. 2) and fern and lycophyte datasets (Fig. 3). In the **first step**, we filtered the original datasets (32,086 angiosperms and 1,253 ferns and lycophytes) for species that occur in the Atlantic Forest domain, resulting in 15,001 angiosperms and 883 ferns and lycophytes. In the **second step**, we searched the resulting list for species identified as occurring on rock outcrops through the “vegetation type” field present in the original datasets, resulting in 1,023 angiosperms and 47 ferns and lycophytes. In the **third step**, we filtered for species that occur in Bahia, Espírito Santo, Minas Gerais and Rio de Janeiro States, corresponding to Sugarloaf Land, resulting in 884 angiosperms and 44 ferns and lycophytes. We conducted the first three steps using the “filter” tool in Microsoft Excel v. 14.5 (Microsoft Office 2010 Proofing Tools).

Finally, in the **fourth step**, we verified the remaining species as those that occur either on lowland inselbergs or other types of rock outcrops, excluding those that occur on highland inselbergs, i.e. above 1000 m a.s.l. (*campos de altitude*), *cangas*, *campo rupestre* and karstic rock outcrops. This verification was based on our own field observations and records of the species in online databases (Jabot-JBRJ 2020, www.jbrj.gov.br/jabot; Reflora - Virtual Herbarium 2020, http://reflora.jbrj.gov.br/reflora/herbarioVirtual/; INCT 2020, http://inct.splink.org.br). We conducted several searches over the period from May 2016 to August 2019 using the keywords “granito”, “granítica”, “granítico”, “gneiss”, “gnáissicos”, “gnaisses”, “inselberg”, “inselbergue”, “incelberg”, “incelbergue”, “pão de açúcar”. At the end of procedure 1, we compiled a list of 208 angiosperms and 16 ferns and lycophytes that are documented as occurring on lowland inselbergs.

In **procedure 2**, we built new datasets for angiosperms (Fig. 2) and ferns and lycophytes (Fig. 3). We combined the species identified in procedure 1 with those sampled by the authors, i.e. species that, to date, have not been documented for lowland inselbergs or were removed during procedure 1 due to incomplete or incorrect information in the original datasets. We also added species based on available literature, such as floristic inventories, original species’ descriptions and ecological studies.

The final checklist is composed of native and non-native plants and includes only vouchers identified to the species level, based on the List of Species of the Brazilian Flora (Flora do Brasil under construction 2020). Correction and updating of the names were performed using the function *get.taxa* in the flora package (Carvalho 2017) for the R software environment (R Core Team 2016), which compares the names in our list with those in the Brazilian Flora online (Flora do Brasil under construction 2020). Families and genera follow Flora do Brasil under construction (2020) and are listed in alphabetical order. The herbaria acronyms are according to Thiers (2020). Vouchers were carefully chosen from specimens collected on vegetation islands and scrub from lowland inselbergs. We added link for the vouchers with available images in Jabot-JBRJ (2020) and Reflora - Virtual Herbarium 2020, which can be continuously updated with regards to taxonomic alterations. A map was built, based on the available coordinates of the vouchers assigned in the lists for angiosperms and ferns and lycophytes in order to ascribe the SLL region. All coordinates were checked to ensure that they occurred on lowland inselbergs; coordinates of municipalities related to the species vouchers were excluded from our sampling. The map was generated in R (R Core Team 2016), using the 'rgdal' and 'concaveman' packages. The first allows the manipulation of shapefiles, the second allows the generation of concave polygons (concave hulls). The Brazilian biomes shapefile came from the Brazilian Ministry of the Environment.

**Habitat types and life forms**: The sampling was restricted to vascular plants that occur in vegetation islands (Porembski et al. 2000), i.e. isolated vegetation patches of various shapes and sizes, bounded by bare rock or directly seated on bare rock (Caiafa and Silva 2005, de Paula et al. 2017, Fig. 4). Another vegetation type, “thicket” or “scrub” (Rizzini 1997, Fig. 4; from hereon, treated as scrub), which occurs in the transition zone between the outcrop and the forest that borders the inselberg, is composed mainly of trees and shrubs from the Atlantic Forest matrix surrounding the inselbergs, with the occasional incidence of species from the vegetation islands. The scrub also includes endemic inselberg species; however, in order to standardise our sampling and filtering in the virtual databases and because of misleading information in the records, we did not include species exclusive to the scrub habitat. Nevertheless, we do include species from vegetation islands that occur occasionally in the scrub, ascribing them to both habitats. We obtained information on life forms for every species, based on the Brazilian Flora online (Flora do Brasil under construction 2020) and classified them into six categories: herbs, lianas/vines, palm trees, shrubs, sub-shrubs and trees.

**Threatened species**: To assign conservation status, we used information from the Centro Nacional de Conservação da Flora (http://www.cncflora.jbrj.gov.br/portal), which provides a continuously-updated list of threatened plant species in the country (MMA 2014).

## Geographic coverage

### Description

The geographic coverage encompasses lowland inselbergs in the States of Rio de Janeiro, Espírito Santo, southern Bahia and eastern Minas Gerais, i.e. Sugarloaf Land (de Paula et al. 2016).

## Taxonomic coverage

### Description

Our final list consisted of a total of 548 species of vascular plants (505 angiosperms; 43 ferns and lycophytes), belonging to 69 families and 212 genera, occurring on lowland inselbergs in the Atlantic Forest in SE Brazil, i.e. Sugarloaf Land. From procedure 1, based originally on BFG (2015)and Prado et al. (2015), we recorded 208 angiosperms and 16 ferns and lycophytes. From procedure 2, which took into account species sampled by the authors (that were not compiled in BFG 2015 and Prado et al. 2015) and those identified in previous published articles, we recorded 297 angiosperms and 27 ferns and lycophytes.

**Figure 5.**
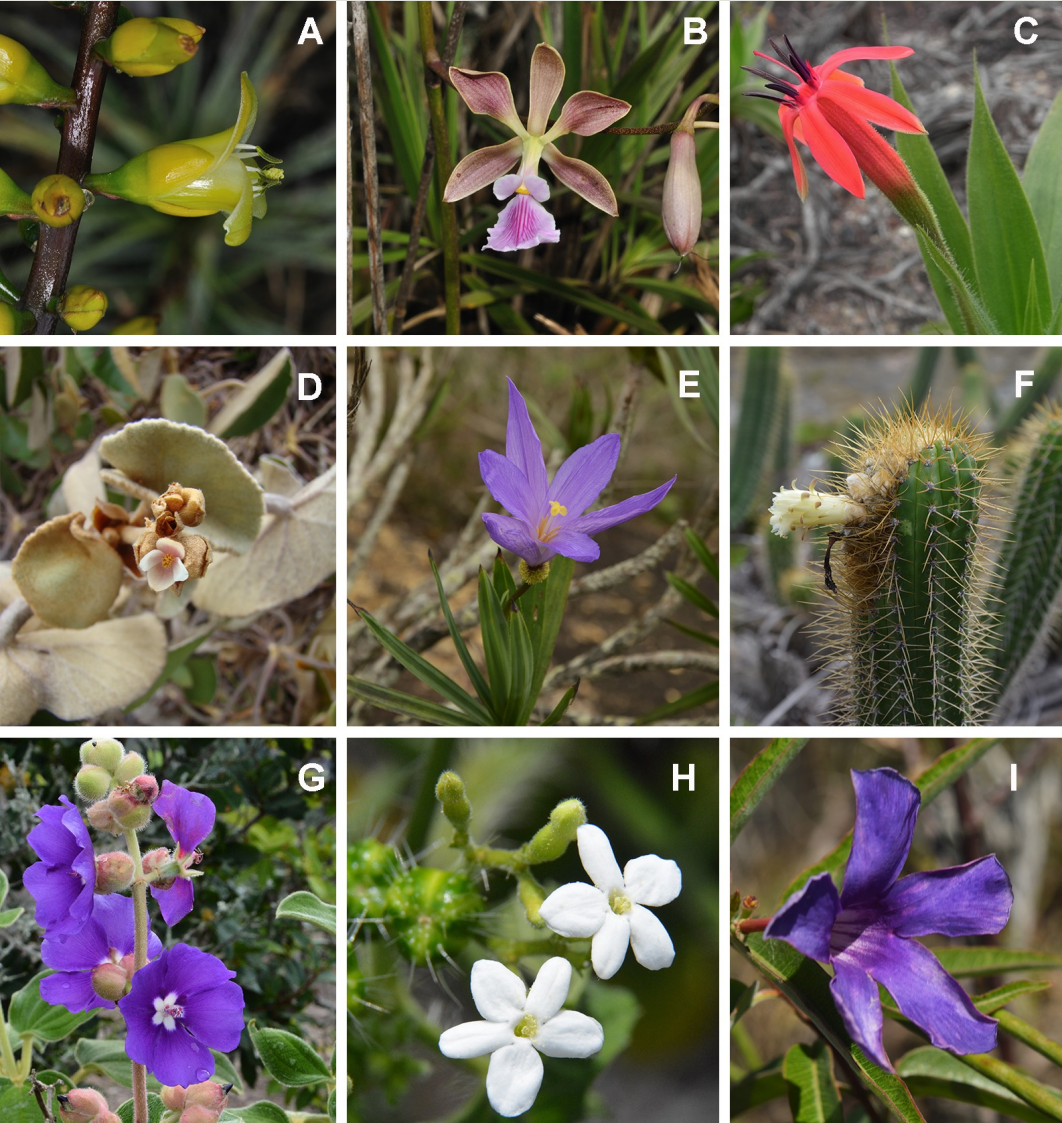
Species belonging to the most representative angiosperm families from vegetation islands occurring on lowland inselbergs in the Atlantic Forest, SE Brazil. **A.** *Encholirium gracile* L.B.Sm . (Bromeliaceae); **B.** *Encyclia spiritusanctensis* L.C.Menezes (Orchidaceae); **C.** *Barbacenia tomentosa* Mart. (Velloziaceae); **D.** *Begonia aguiabrancensis* L.Kollmann (Begoniaceae); **E.** *Vellozia pulchra* L.B.Sm. (Velloziaceae); **F.** *Coleocephalocereus fluminensis* (Miq.) Backeb. (Cactaceae); **G.** *Pleroma marinanum* P.J.F. Guim. & Fraga (Melastomataceae); **H.** *Cnidoscolus lombardii* Fern.Casas (Euphorbiaceae); **I.** *Mandevilla grazielae* M.F.Sales *et al.* (Apocynaceae). Photos A, B, D, E, I by L.F.A. de Paula; C, F, G, H by L.O. Azevedo.

**Figure 6.**
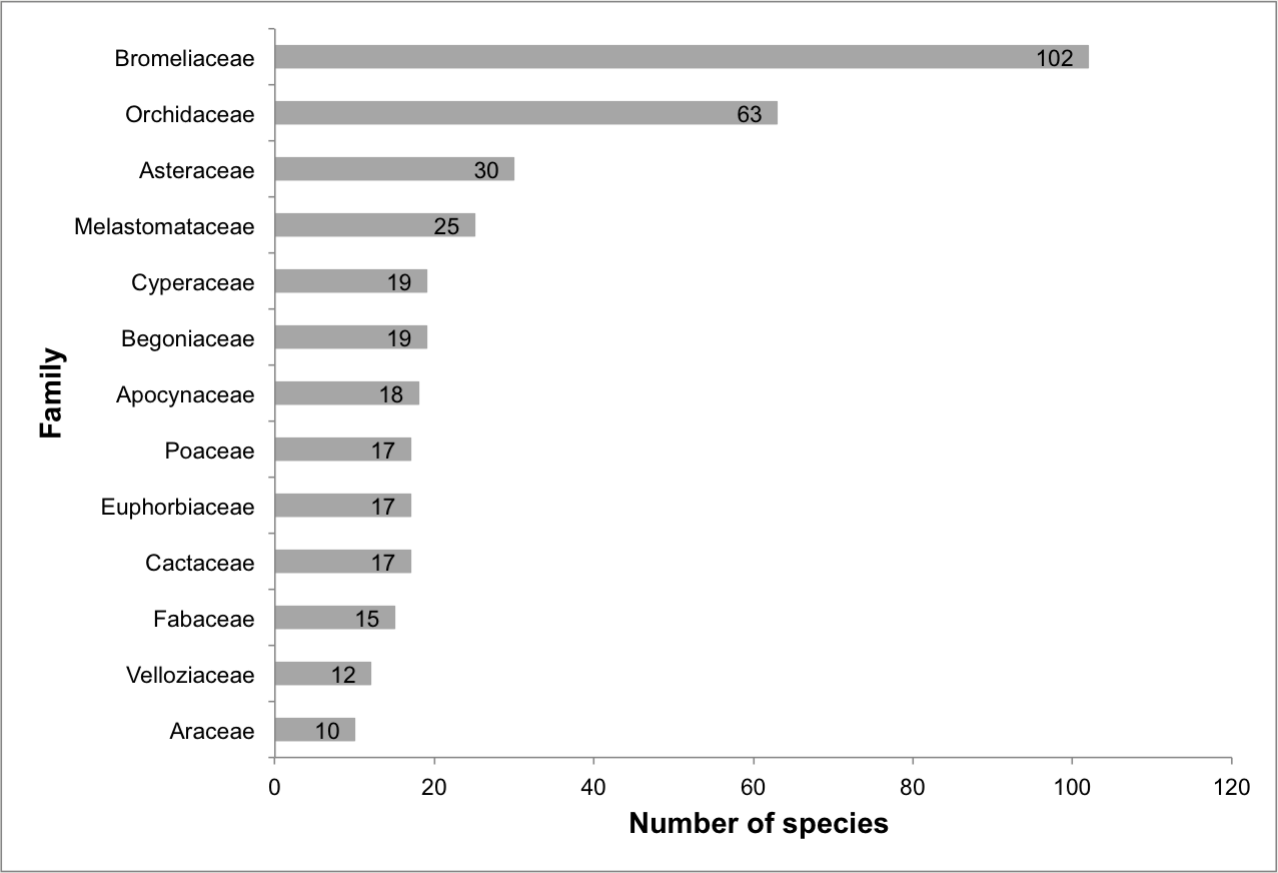
Richest angiosperm families from lowland inselbergs in the Atlantic Forest, SE Brazil.

**Figure 7.**
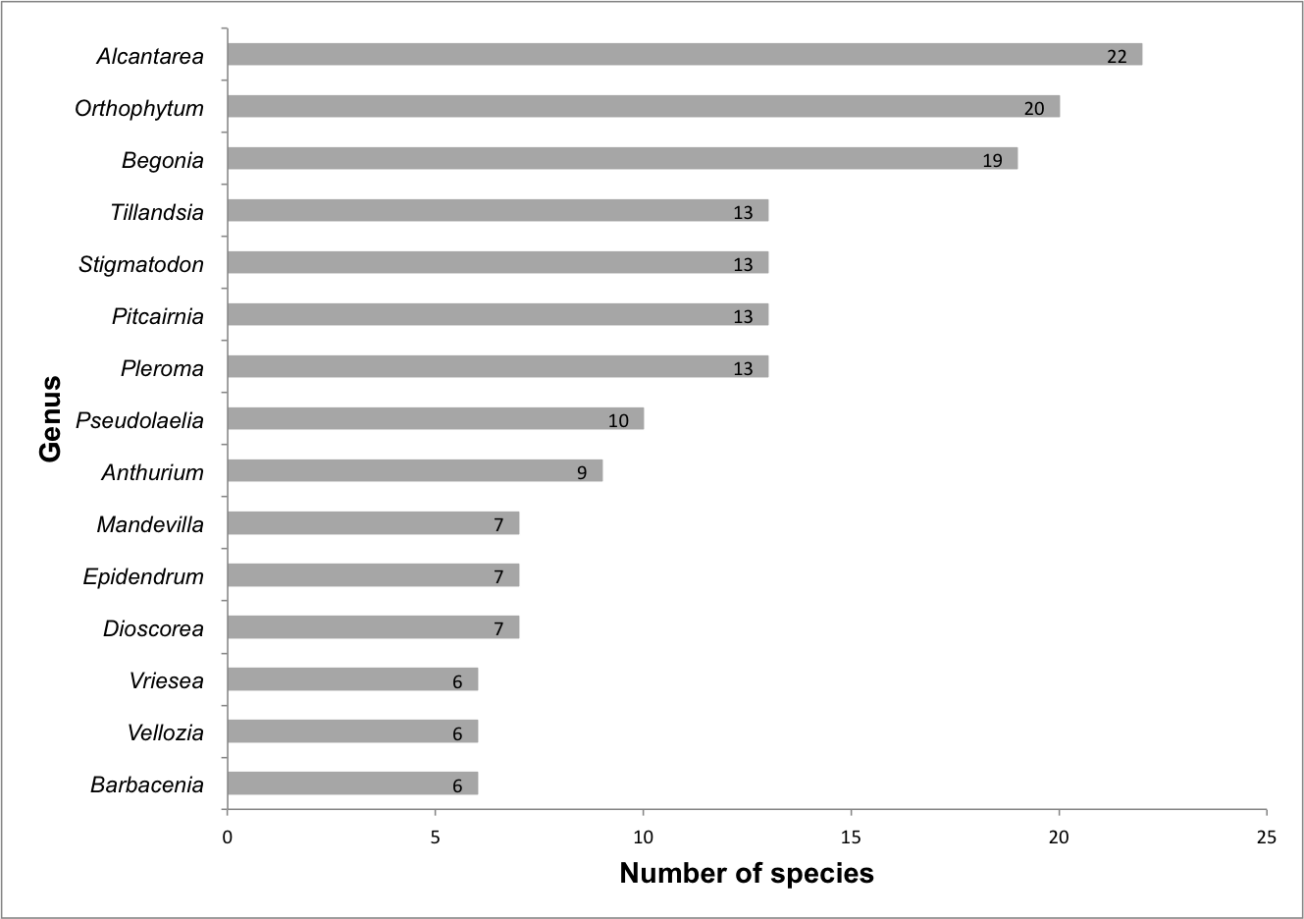
Richest angiosperm genera from lowland inselbergs in the Atlantic Forest, SE Brazil.

We identified a total of 505 species of angiosperms (493 species are native and 12 are naturalised) belonging to 58 families and 192 genera (Suppl. material 1; Fig. 5). The richest families are Bromeliaceae (102 spp.), Orchidaceae (63 spp.), Asteraceae (30 spp.), Melastomataceae (25 spp.), Begoniaceae (19 spp.), Cyperaceae (19 spp.), Apocynaceae (18 spp.), Cactaceae (17 spp.), Euphorbiaceae (17 spp.), Poaceae (17 spp.), Fabaceae (15 spp.), Velloziaceae (12 spp.) and Araceae (10 spp.) (Fig. 6). Sixteen families are represented by only one species. The richest genera are *Alcantarea* (22 spp.), *Orthophytum* (20 spp.), *Begonia* (19 spp.), *Pitcairnia* (13 spp.), *Pleroma* (13 spp.), *Stigmatodon* (13 spp.), *Tillandsia* (13 spp.), *Pseudolaelia* (10 spp.), *Anthurium* (9 spp.), *Dioscorea* (7 spp.), *Epidendrum* (7 spp.), *Mandevilla* (7 spp.), *Barbacenia* (6 spp.), *Vellozia* (6 spp.) and *Vriesea* (6 spp.) (Fig. 7). A total of 96 genera are represented by only one species.

**Figure 8.**
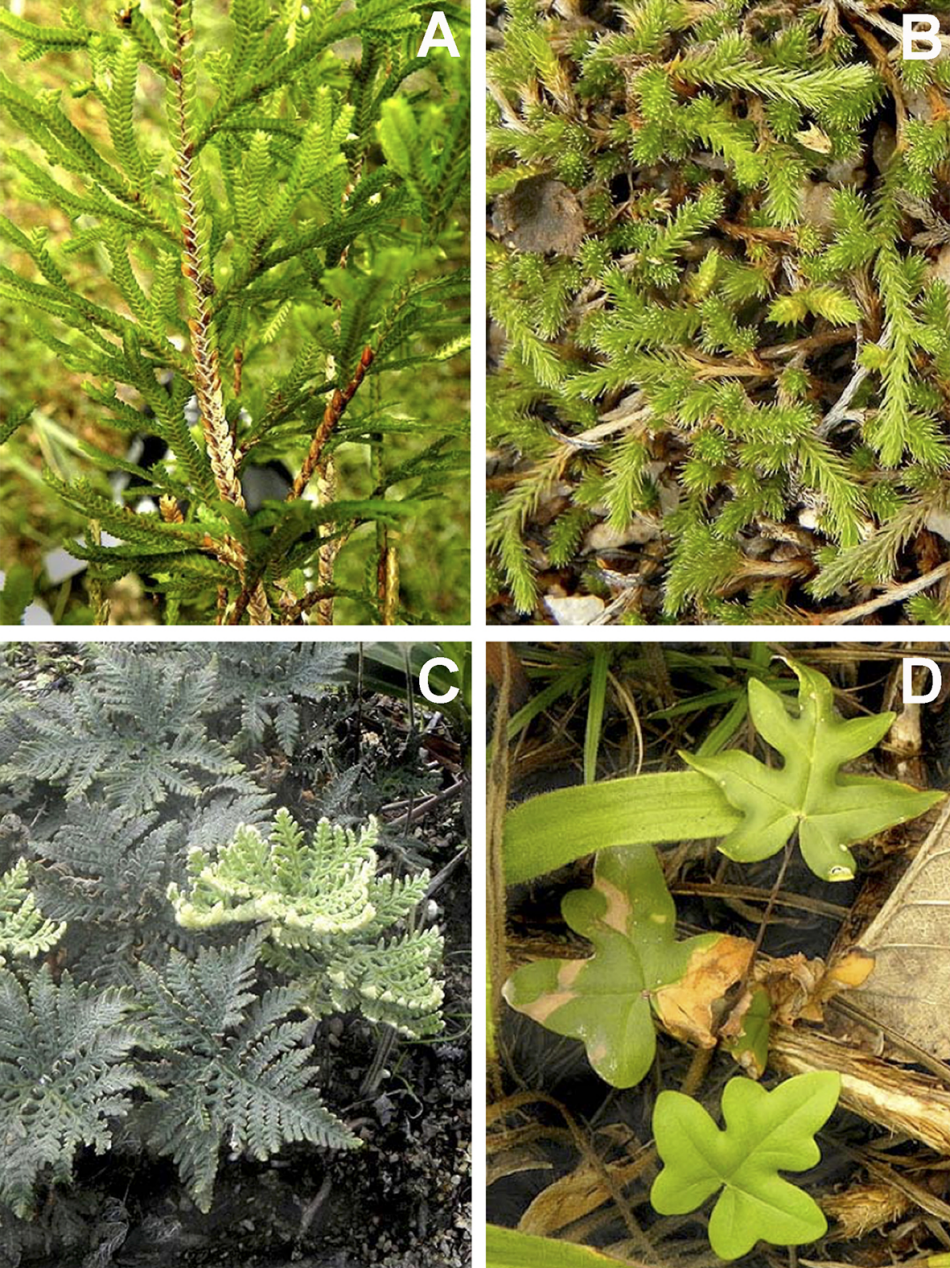
Species belonging to the most representative fern and lycophyte families from vegetation islands occurring on lowland inselbergs in the Atlantic Forest, SE Brazil. **A.** *Selaginella convoluta* (Arn.) Spring (Selaginellaceae); **B.** *S. sellowii* Hieron. (Selaginellaceae); **C.** *Cheilanthes geraniifolia* (Weath.) R.M.Tryon & A.F.Tryon (Pteridaceae); **D.** *Doryopteris collina* (Raddi) J.Sm. (Pteridaceae).

**Figure 9.**
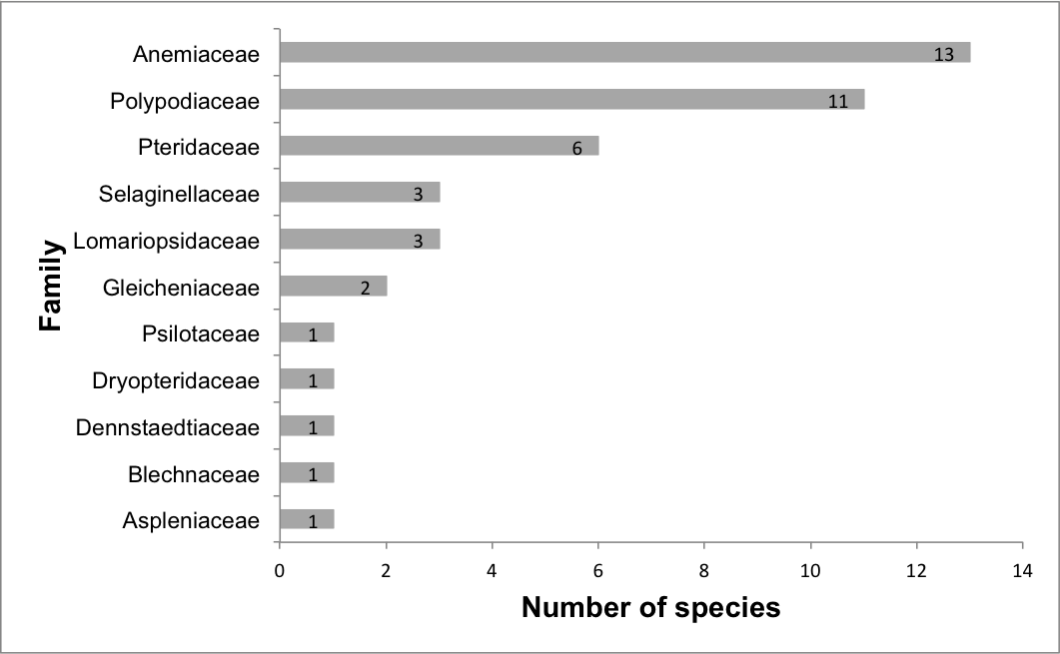
Richest fern and lycophyte families from lowland inselbergs in the Atlantic Forest, SE Brazil.

**Figure 10.**
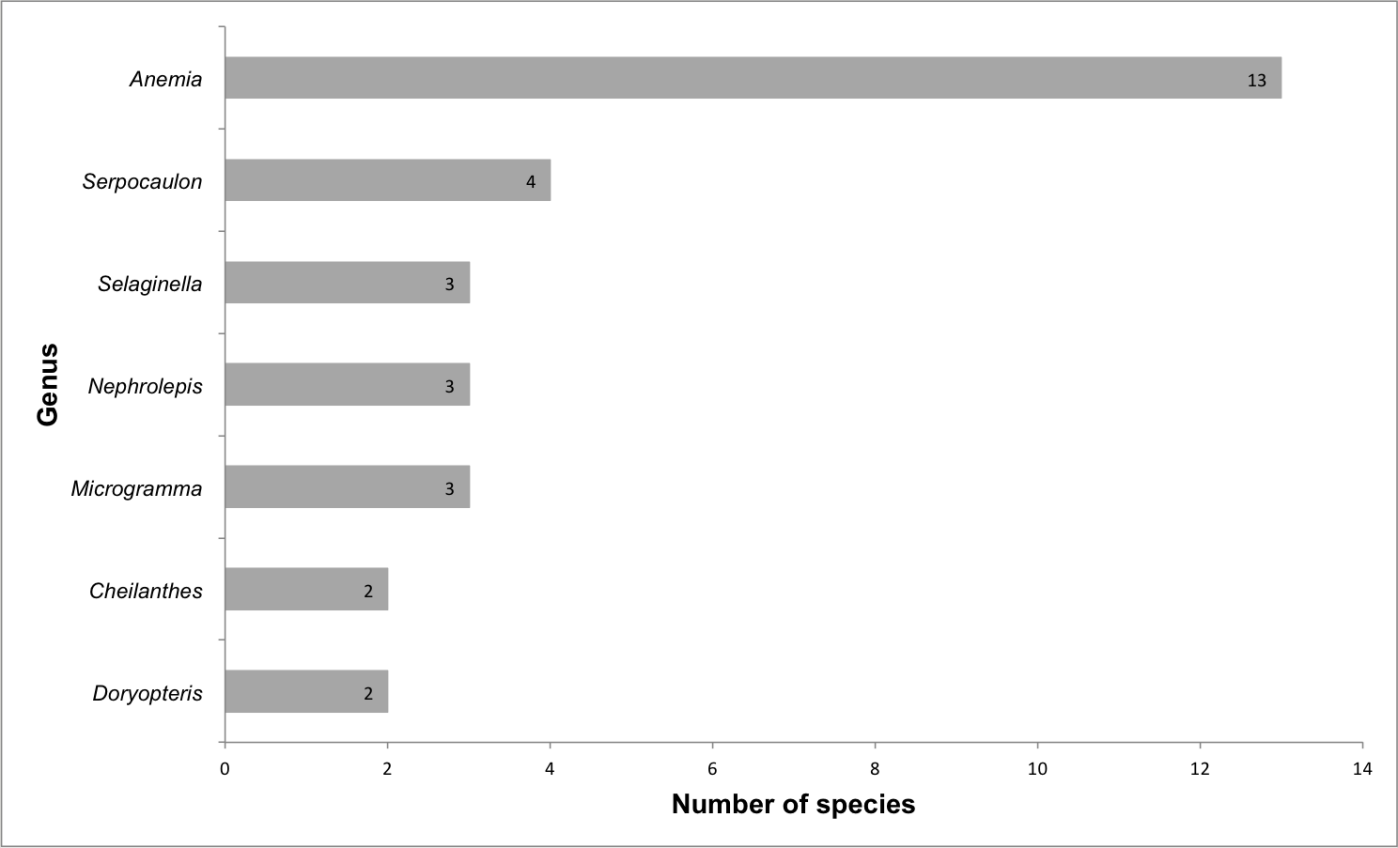
Richest fern and lycophyte genera from lowland inselbergs in the Atlantic Forest, SE Brazil.

We recorded a total of 43 species of ferns and lycophytes (all species are native) belonging to 11 families and 20 genera (Suppl. material 2; Fig. 8). The richest families are Anemiaceae (13 spp.), Polypodiaceae (11 spp.), Pteridaceae (6 spp.), Selaginellaceae (3 spp.), Lomariopsidaceae (3 spp.) and Gleicheniaceae (2 spp.) (Fig. 9). Five families are represented by only one species. The richest genera are *Anemia* (13 spp.), *Serpocaulon* (4 spp.), *Microgramma* (3 spp.), *Nephrolepis* (3 spp.), *Selaginella* (3 spp.), *Doryopteris* (2 spp.) and *Cheilanthes* (2 spp.) (Fig. 10). There were 13 genera presenting only one species.

**Life forms and habitat types**

**Figure 11.**
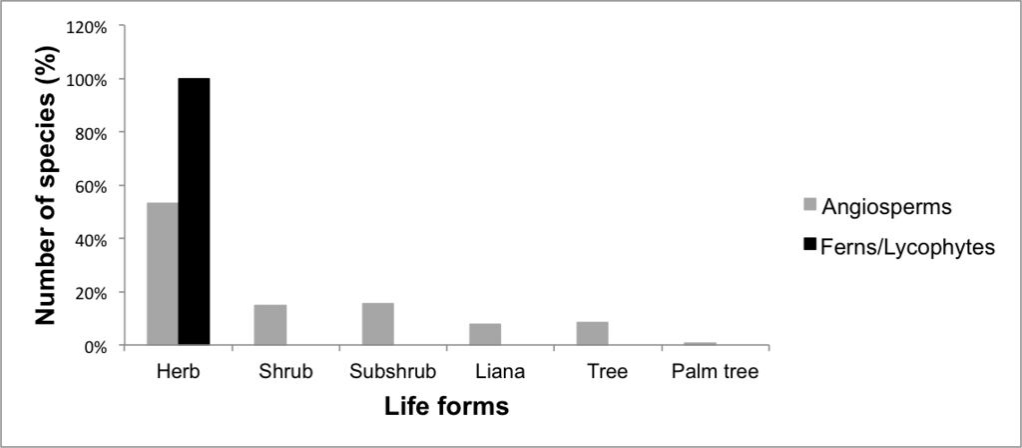
Life forms of the vascular plant species occurring on lowland inselbergs in the Atlantic Forest, SE Brazil.

**Figure 12.**
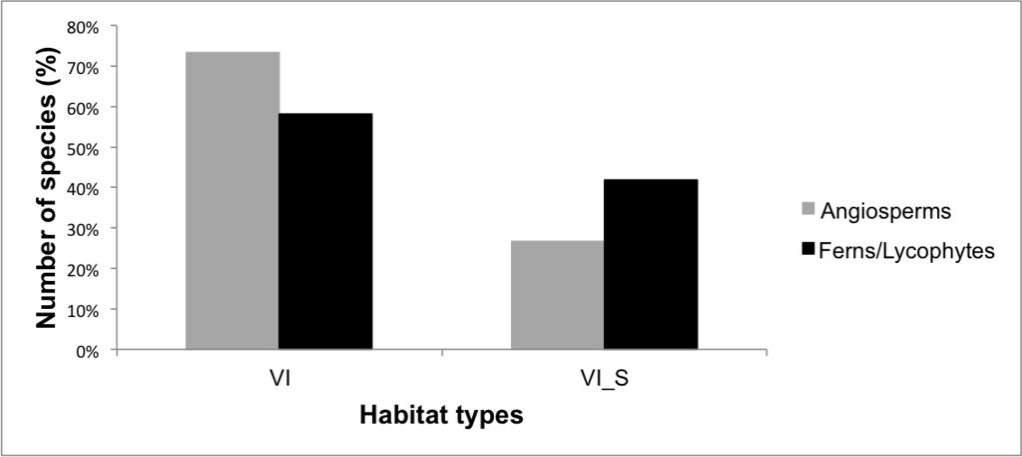
Percentage of vascular plant species occurring in different habitat types on lowland inselbergs in the Atlantic Forest, SE Brazil. VI = vegetation islands; VI_S = vegetation islands and scrub.

We determined that 53% (268 spp.) of angiosperms are herbs, followed by shrubs (15%; 75 spp.), subshrubs (15%; 78 spp.), lianas (8%; 39 spp.), trees (8%; 42 spp.) and palm trees (1%; 3 spp.) (Fig. 11). All species of ferns and lycophytes are herbs (Fig. 11). Most of the species (73% of the angiosperms and 58% of the ferns and lycophytes) occurred in vegetation islands, but there were species which occurred in both vegetation islands and scrub (27% of angiosperms and 42% of ferns and lycophytes) (Fig. 12).

**Vegetation type, vouchers, conservation status**

**Figure 13.**
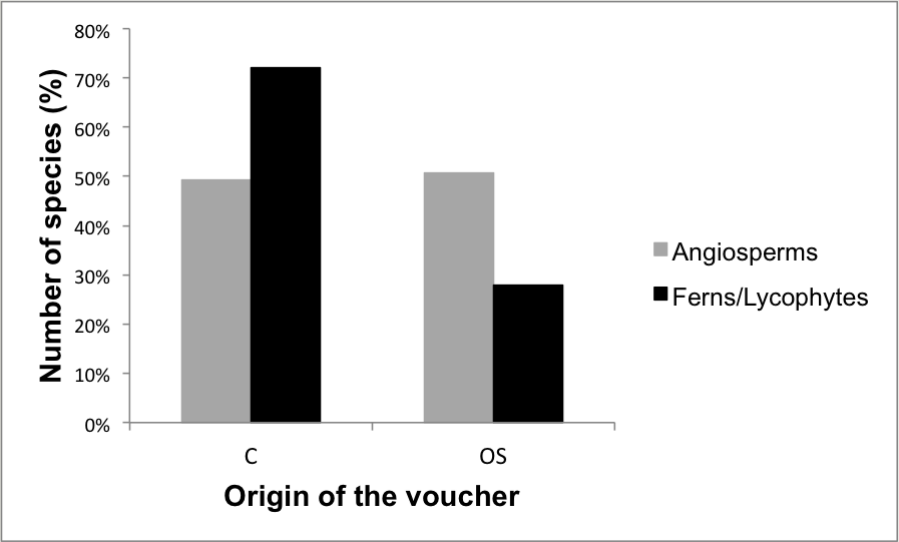
Percentages of the vascular plant species included in the checklist that were collected by the authors (C) or vouchered indirectly from other sources (OS).

A total of 59% of the angiosperms and 63% of the ferns and lycophytes on our final list were not described as occurring on “rock outcrops” in BFG (2015) and Prado et al. (2015), respectively. Therefore, this study helps to better define information in the "vegetation type" field in Flora do Brasil under construction (2020). Furthermore, 49% (249 spp.) of the angiosperms and 72% (31 spp.) of the ferns and lycophytes were collected by the authors (Fig. 13), the remaining species being vouchered indirectly from other sources. As a first step to generate a Virtual Herbarium of the vascular plants of Sugarloaf Land, we added links with available images for the vouchers for 75% of the total number of species (375 angiosperms and 37 ferns and lycophytes). The advantage of the link, besides enabling the reader to see the image of the voucher with the respective original collection label, is that taxonomic changes can be revised in the databases, which allows the list to be continuously updated. In the official lists of endangered species, 115 angiosperms and five ferns and lycophytes are mentioned. In the angiosperm list, 9 species are vulnerable (VU), 30 are endangered (EN) and 12 are critically endangered (CR), while in the fern and lycophyte list, two species are vulnerable; the remaining species are in least concern categories (see Suppl. materials 1, 2). However, the conservation status of most of the angiosperm (77%), fern and lycophyte (88%) species is unknown.

**Sugarloaf Land region**

**Figure 14.**
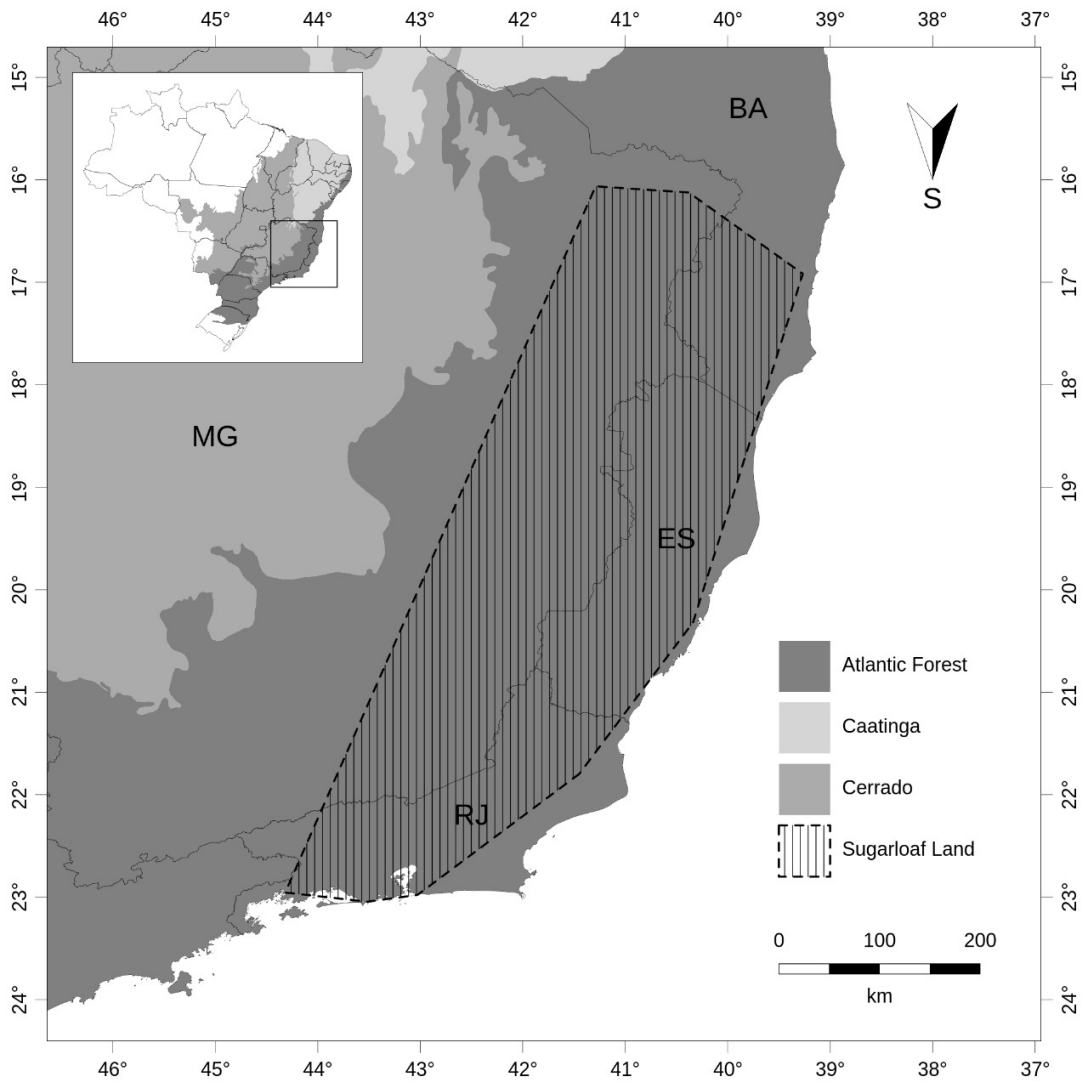
General overview of Sugarloaf Land (SLL) region. The concave polygon that indicates SLL was built, based on the available coordinates of the vouchers assigned in the vascular plant list for lowland inselbergs occurring in the Atlantic Forest, SE Brazil.

SLL region was originally baptised, based on projections generated through modelling techniques only for the Bromeliaceae family (de Paula et al. 2016) and here we aimed to cover the occurrence of all the vascular plant species occurring on lowland inselbergs. The convex envelope representing the SLL region is displayed in Fig. 14 and contains the records for lowland inselbergs where we had access to the coordinates. It is important to note that lowland inselbergs might exist outside the area ascribed here as SLL, due to the following facts: i) many lowland inselbergs have never been sampled; ii) some vouchers did not have available coordinates; these factors culminate in the absence of possible lowland inselberg areas in our map. However, it gives a better estimation and a finer overview of the core area named as SLL.

## Usage rights

### Use license

Creative Commons Public Domain Waiver (CC-Zero)

## Data resources

### Data package title

Sugarloaf Land in south-eastern Brazil: a tropical hotspot of lowland inselberg plant diversity - Supplementary Material

### Resource link

https://ckan.jbrj.gov.br/dataset/2020_bdj_inselberg

### Number of data sets

2

### Data set 1.

#### Data set name

List of angiosperms occurring on lowland inselbergs in the Atlantic Forest, SE Brazil

#### Data format

CSV and XLSX

#### Number of columns

10

#### Download URL

https://ckan.jbrj.gov.br/dataset/2020_bdj_inselberg

#### Description

List containing 505 angiosperm species occurring on lowland inselbergs in the Atlantic Forest, SE Brazil, highlighting species included in official lists of endangered flora, life forms, habitat type of occurrence, origin, voucher, link for the herbarium image of the voucher.

**Data set 1.** 

Column label	Column description
Family	The full scientific name of the family in which the taxon is classified
Species	The full scientific name
Author	Authorship of the scientific name
Origin	If the species is native or non-native, followed Flora do Brasil 2020 (under construction; http://floradobrasil.jbrj.gov.br)
Threatened category	Followed Centro Nacional de Conservação da Flora (http://www.cncflora.jbrj.gov.br/portal); DD = Data deficient, NT = Near Threatened, VU = Vulnerable, CR = Critically Endangered, EN = Endangered, LC = Least Concern, NE = Not evaluated
Life form	Followed Flora do Brasil 2020 (under construction; http://floradobrasil.jbrj.gov.br); life forms are classified into six categories: herbs, lianas/vines, palm trees, shrubs, sub-shrubs and trees
Source	Indicates where we achieved the information that the species is occurring on lowland inselbergs
Habitat	Indicates if the species occurred in vegetation islands (VI; ellipsoid vegetation patches surrounded by bare rock) and scrub (S; arboreal-shrub vegetation bordering inselberg)
Reference or voucher	Indicates vouchers (collector and number) with the respective acronym of the herbarium according to Thiers (http://sweetgum.nybg.org/ih/, continuously updated) when the source is C or EC; in case the source is A, it was provided by the reference of the article (see references at the end of the table)
Link for the voucher	Provides the link for the online image of the respective voucher; links are from JABOT-JBRJ and Reflora-Herbário Virtual

### Data set 2.

#### Data set name

List of ferns and lycophytes occurring on lowland inselbergs in the Atlantic Forest, SE Brazil

#### Data format

CSV and XLSX

#### Number of columns

10

#### Download URL

https://ckan.jbrj.gov.br/dataset/2020_bdj_inselberg

#### Description

List containing 43 fern and lycophyte species occurring on lowland inselbergs in the Atlantic Forest, SE Brazil, highlighting species included in official lists of endangered flora, life forms, habitat type of occurrence, origin, voucher, link for the herbarium image of the voucher.

**Data set 2.** 

Column label	Column description
Family	The full scientific name of the family in which the taxon is classified
Species	The full scientific name
Author	Authorship of the scientific name
Origin	If the species is native or non-native, followed Flora do Brasil 2020 (under construction; http://floradobrasil.jbrj.gov.br)
Threatened category	Followed Centro Nacional de Conservação da Flora (http://www.cncflora.jbrj.gov.br/portal); DD = Data deficient, NT = Near Threatened, VU = Vulnerable, CR = Critically Endangered, EN = Endangered, LC = Least Concern, NE = Not evaluated
Life form	Followed Flora do Brasil 2020 (under construction; http://floradobrasil.jbrj.gov.br); life forms are classified into six categories: herbs, lianas/vines, palm trees, shrubs, sub-shrubs and trees
Source	Indicates where we achieved the information that the species is occurring on lowland inselbergs
Habitat	Indicates if the species occurred in vegetation islands (VI; ellipsoid vegetation patches surrounded by bare rock) and scrub (S; arboreal-shrub vegetation bordering inselberg)
Reference or voucher	Indicates vouchers (collector and number) with the respective acronym of the herbarium according to Thiers (http://sweetgum.nybg.org/ih/, continuously updated) when the source is C or EC; in case the source is A, it was provided by the reference of the article (see references at the end of the table)
Link for the voucher	Provides the link for the online image of the respective voucher; links are from JABOT-JBRJ and Reflora-Herbário Virtual

## Additional information

**Checklist, ecological aspects and implications for conservation**

The beta diversity of lowland inselbergs in Brazil is outstanding (Meirelles et al. 1999, Safford and Martinelli 2000, de Paula et al. 2016, de Paula et al. 2019b), with each outcrop containing exclusive flora, even though they may be located only a few kilometres apart. The high species turnover across individual inselbergs is a common pattern worldwide, for instance, it has been shown for inselberg mosaics in Australia (Yates et al. 2019) and Northern South America (Sarthou et al. 2017). Moreover, studies on neotropical inselberg-adapted species have shown high population differentiation, high genetic diversity levels and strong phylogeographic structure in this naturally-fragmented environment (e.g. Barbará et al. 2007, Palma-Silva et al. 2011, Hmeljevski et al. 2015, Hmeljevski et al. 2017, de Paula et al. 2017b, Nazareno et al. 2020). Species with patchy distribution usually experience reduced gene flow, significant genetic drift and high levels of population divergence, supporting the view of inselbergs as centres of species diversity and endemism.

Furthermore, a common phenomenon on these outcrops is the occurrence of polymorphic species (Cronk 1998, Mello-Silva 2004). Many inselberg taxa have previously been described as displaying extensive intraspecific variation amongst and within populations, especially monocot taxa, such as *Anthurium*, *Philodendron* (Araceae), *Alcantarea*, *Encholirium*, *Pitcairnia* (Bromeliaceae), *Pseudolaelia* (Orchidaceae), *Barbacenia* and *Vellozia* (Velloziaceae), but also eudicot taxa, such as *Pleroma* (Melastomataceae) and *Coleocephalocereus* (Cactaceae) (Mello-Silva 2004, Taylor and Zappi 2004, Hmeljevski et al. 2017, de Paula et al. 2017a, Menini Neto et al. 2019). The spatial and ecological isolation of these outcrops as a result of their immersion in the surrounding rainforest is thought to be responsible for the high levels of genetic differentiation amongst populations, including *Alcantarea* (Barbará et al. 2007), *Encholirium* (Hmeljevski et al. 2015, Hmeljevski et al. 2017) and *Pitcairnia* (Palma-Silva et al. 2011), as well as for the resulting morphological variability commonly reported in adaptive radiation events (Palma-Silva et al. 2011). In developing the present checklist, we could not provide information on species complexes, but note that they should be taken into consideration in conservation analyses since outcrops harbour specific morphotypes. Attention to these processes is also important for solving taxonomic problems and understanding processes of species evolution within terrestrial islands.

The high levels of species diversity on inselbergs is usually linked to the fact that species capable of surviving in such harsh environmental conditions display diverse forms of adaptation (Biedinger et al. 2000, de Paula et al. 2015, de Paula et al. 2019a). With regard to water scarcity, distinct plant groups in SE Brazil have adopted several strategies to mitigate such stresses. The succulent leaves of bromeliads of the genus *Encholirium* and orchids of the genus *Epidendrum*, for example, enable plants to accumulate water in their tissue. Other bromeliads, such as *Alcantarea* spp. and *Vriesea* spp., can store water in reservoirs due to their rosette-shaped leaf arrangement, which also attracts a variety of fauna (Lacerda et al. 2009). Cacti, in turn, not only accumulate water, but also have spines (modified leaves) and trichome-like thorns/spines at the stem base, which enables them to avoid overheating when lying directly on the rock, an adaption typical of *Coleocephalocereus* species. Geophytes that store water in underground bulbs are also very common, such as members of the Apocynaceae family (e.g. *Mandevilla* spp.). In contrast, desiccation tolerance is an effective strategy used by some inselberg species to cope with drought conditions, for instance, present in Velloziaceae members (e.g. *Barbacenia* spp. and *Vellozia* spp.), but also in fern and lycopyte species (e.g. *Cheilanthes* spp., *Doryopteris* spp., *Selaginella* spp.). This strategy is characterised by the ability of plants to survive cycles of dehydration and rehydration without affecting viability (Porembski and Barthlott 2000). During the dry season, desiccation-tolerant species enter a dehydrated state, losing a considerable amount of water in their tissue. With the occurrence of rain, they quickly rehydrate, in many cases without any structural or physiological damage (Vicré et al. 2004). Due to this unique feature, they are called resurrection plants. Understanding the heterogeneity of ecological strategies found amongst inselberg species can be useful in developing conservation strategies, for example, for restoration after disturbance, especially in the context of global environmental change (Drenovsky et al. 2012, de Paula et al. 2015, Ottaviani et al. 2016). Since species have different levels of tolerance to abiotic stresses, conservationists can select species based on their traits, which includes attributes associated with resource acquisition and growth and their functional role (Tecco et al. 2010, de Paula et al. 2015).

Due to difficulties in accessing many inselbergs, researchers concerned with biodiversity have overlooked the significant potential of these outcrops. The high levels of beta diversity and genetically-differentiated populations on outcrops in SE Brazil underscore the fact that there are insufficient numbers of inselbergs protected within conservation units. Furthermore, lowland inselbergs are most often located in small reserves within urban areas, such as the *Monumento Natural dos Morros do Pão de Açúcar e da Urca*, the *Parque Nacional da Tijuca*, the *Parque Natural Municipal Paisagem Carioca Rio de Janeiro* and the *Parque Estadual Serra da Tiririca*, all of which are located within the metropolitan area of Rio de Janeiro. In these reserves, inselbergs can be visited by tourists, which can have a significant impact on the natural vegetation. There are also some lowland inselbergs located in conservation units which permit a certain amount of human occupation, for example, the *Pedra do Elefante* in Espírito Santo State (Pena and Alves-Araújo 2017). The *Monumento Natural Pontões Capixabas*, in the heart of the Sugarloaf Land in Espírito Santo, is the only conservation area in Brazil that encompasses a considerable number of lowland inselbergs. However, its effectiveness is threatened by the absence of a reasonable management plan (http://www.icmbio.gov.br/portal/planosmanejo) that could help to balance the competing interests of local farmers with the preservation of its unique biota.

In Brazil, threats to inselberg biodiversity is increasing dramatically due to quarrying, mining, grazing, goat herding, fire, biological invasions, urban expansion and unsustainable removal of attractive floral species (Martinelli 2007, Porembski et al. 2016, Fig. 15). In Sugarloaf Land, not only is the extraction of natural resources far too vast to be sustainable (Ferreira et al. 2014), but the area is also experiencing rapid fragmentation of the Atlantic Forest biome (Tabarelli et al. 2004), both of which increase the risks to vegetation refugia in inselbergs. In several instances, rocks have taken the place of wood as building and finishing material and the destructive cycle of forest logging (Dean 1995) has been replaced by the extraction of geological resources, once again fuelling an uncontrolled and unsustainable industry. The lack of conservation units that harbour inselbergs demonstrates the need for inventories of those that occur on private lands. Meanwhile, taxonomically-verified checklists are not only necessary for ecological, genetic, biogeographic and evolutionary studies (Cardoso et al. 2017), but also to provide information for Brazilian policies of biological conservation that include rock outcrop ecosystems. Correct estimates of the flora provide reliable data and information to promote the activities necessary to preserve these extremely threatened environments. Finally, as populations on individual inselbergs are genetically distinct and beta diversity between outcrops is extremely high, effective conservation strategies will face serious challenges to protect multiple inselbergs across the whole Sugarloaf Land region.

## Supplementary Material

AFC64366-6587-5D9F-8BD4-1FC80ACCBFAD 10.3897/BDJ.8.e53135.suppl1 Supplementary material 1 List of angiosperms occurring on lowland inselbergs in the Atlantic Forest, SE Brazil Data type List of species Brief description List containing 505 angiosperm species occurring on lowland inselbergs in the Atlantic Forest, SE Brazil, highlighting species included in official lists of endangered flora, life forms, habitat type of occurrence, origin, voucher, link for the herbarium image of the voucher. File: oo_415843.csv https://binary.pensoft.net/file/415843 de Paula, LFA; Azevedo, LO; Mauad, LP; Cardoso, LJT; Braga, JMA; Kollmann, LJC; Fraga, CN; Menini Neto, L; Labiak, PH; Mello-Silva, R; Porembski, S; Forzza, RC

E942A671-3BE3-5D84-B429-2AF2E1C50B70 10.3897/BDJ.8.e53135.suppl2 Supplementary material 2 List of ferns and lycophytes occurring on lowland inselbergs in the Atlantic Forest, SE Brazil Data type List of species Brief description List containing 43 fern and lycophyte species occurring on lowland inselbergs in the Atlantic Forest, SE Brazil, highlighting species included in official lists of endangered flora, life forms, habitat type of occurrence, origin, voucher, link for the herbarium image of the voucher. File: oo_415853.csv https://binary.pensoft.net/file/415853 de Paula, LFA; Azevedo, LO; Mauad, LP; Cardoso, LJT; Braga, JMA; Kollmann, LJC; Fraga, CN; Menini Neto, L; Labiak, PH; Mello-Silva, R; Porembski, S; Forzza, RC

## Figures and Tables

**Figure 4.**
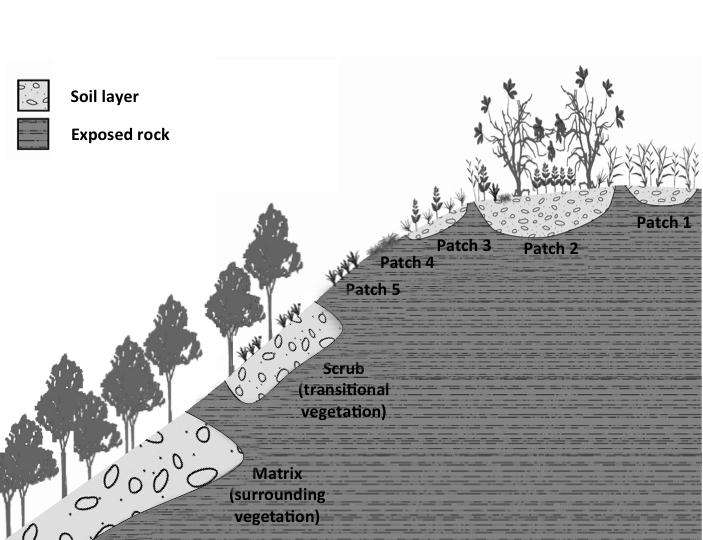
Schematic representation of inselberg vegetation. Vegetation islands are represented by patches 1-5 of varying shapes and sizes, which are bounded by bare rock or directly seated on bare rock (Porembski et al. 2000). Scrub vegetation (Rizzini 1997) appears in the transition zone between the outcrop and the forest that borders the inselberg. The matrix is characterised by the surrounding vegetation, in this case represented by the forest physiognomies belonging to the Atlantic Forest domain. The schematic representation was adapted from de Paula et al. (2015).

**Figure 15.**
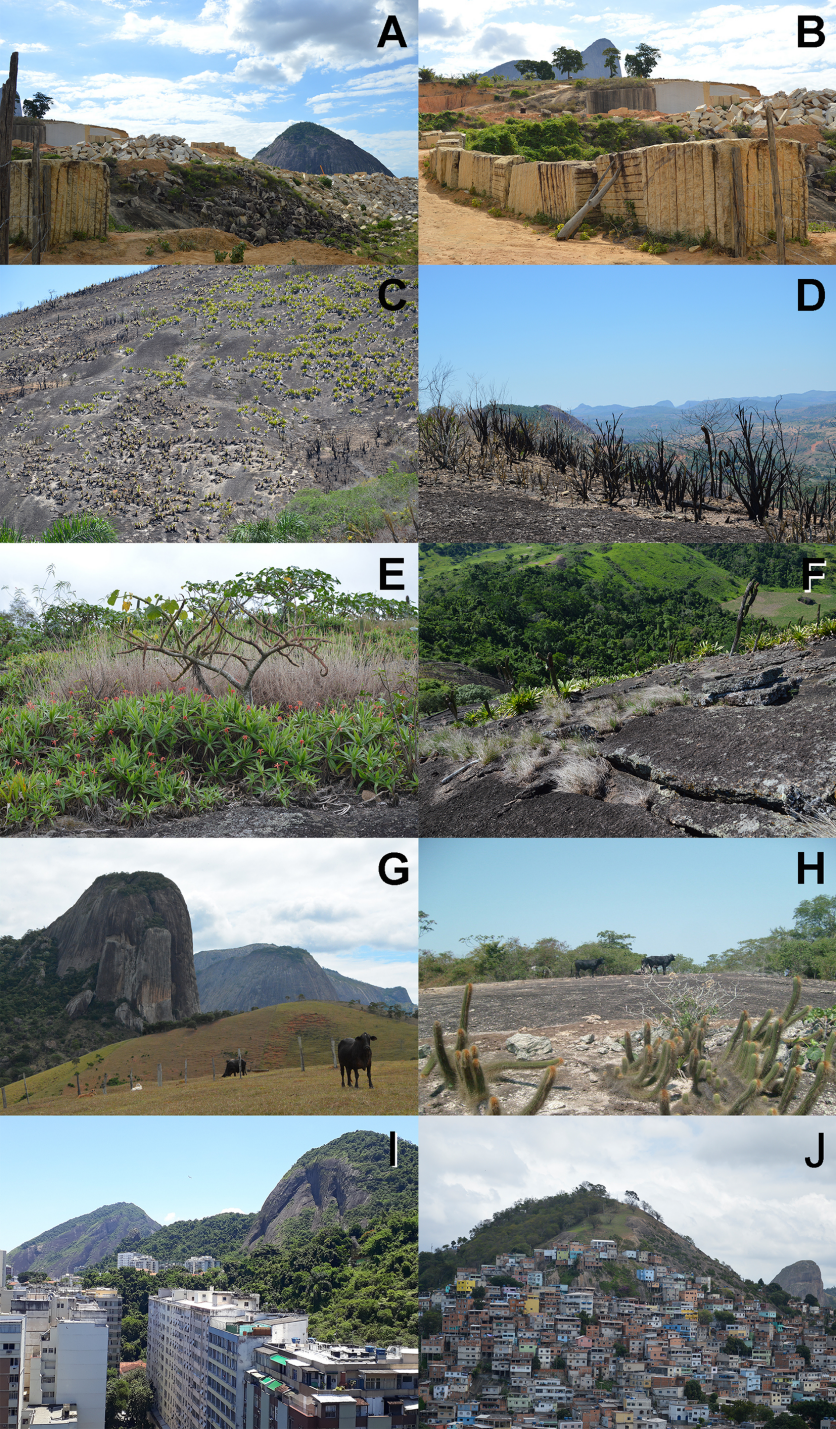
Main threats to inselberg biodiversity in SE Brazil. **A, B.** Mining in Espírito Santo State; **C, D.** Burned populations of *Alcantarea extensa* (Bromeliaceae) and *Vellozia plicata* (Velloziaceae), respectively, due to anthropogenically-caused fire in Minas Gerais State; **E, F.** Invasive grass (*Melinis repens*) on inselbergs in Minas Gerais State; **G, H.** Grazing on inselberg surroundings and on the top of an inselberg, respectively, in Minas Gerais State; **I, J.** Urban expansion on to inselbergs in the municipalities of Rio de Janeiro (Rio de Janeiro State) and Vitória (Espírito Santo State), respectively. Photos by L.F.A. de Paula, except for photo I by J.M.A. Braga.
